# MicroRNA-7 inhibits cell proliferation, migration and invasion in human non-small cell lung cancer cells by targeting FAK through ERK/MAPK signaling pathway

**DOI:** 10.18632/oncotarget.12684

**Published:** 2016-10-15

**Authors:** Qi Cao, Zheng-Dao Mao, Yu-Jia Shi, Yi Chen, Yun Sun, Qian Zhang, Lei Song, Li-Ping Peng

**Affiliations:** ^1^ Department of Respiratory Medicine, Affiliated Changzhou No.2 People's Hospital, Nanjing Medical University, Changzhou 213003, P.R. China

**Keywords:** non-small cell lung cancer, microRNA-7, FAK, ERK/MAPK signaling pathway, cell proliferation

## Abstract

**Objective:**

To investigate the effects of microRNA-7 (miR-7) on the proliferation, migration and invasion of non-small cell lung cancer NSCLC) cells by targeting FAK through ERK/MAPK signaling pathway.

**Methods:**

NSCLC tissues and adjacent normal tissues were obtained from 160 NSCLC patients after operation. NSCLC cell lines (A549, H1299 and H1355) and a normal human fetal lung fibroblast cell line (MRC-5) were obtained. NSCLC cells were assigned into miR-7 inhibitors, miR-7 mimics, blank, miR-7 mimics control, miR-7 inhibitors control, FAK siRNA and miR-7 inhibitors + FAK siRNA groups. The expressions of miR-7 and FAK mRNA in tissues and cell lines were detected by qRT-PCR and Western-Blotting. Cell proliferation, migration and invasion were detected by MTT assay, wound scratch assay and Transwell assay.

**Results:**

Compared with adjacent normal tissues, miR-7 expression was down-regulated, but the mRNA and protein expressions of FAK, ERK and MAPK were up-regulated. Compared with the blank and mimics control groups, miR-7 significantly increased but FAK, ERK and MAPK expressions decreased in miR-7 mimics and FAK siRNA groups. Cell proliferation, migration and invasion were inhibited in the miR-7 mimics and FAK siRNA groups, while opposite regarding miR-7 inhibitors group.

**Conclusion:**

The miR-7 can inhibit the activation of ERK/MAPK signaling pathway by down-regulating FAK expression, thereby suppressing the proliferation, migration and invasion of NSCLC cells. The miR-7 and its target gene FAK may be novel targets for the diagnosis and treatment of NSCLC.

## INTRODUCTION

Lung cancer remains a major worldwide health problem, comprising 17% of the total new cancer cases and 23% of the total cancer deaths, tobacco epidemic, second-hand smoke and environmental pollution increase incidence and mortality burden [1, 2]. Importantly, non-small cell lung cancer (NSCLC) accounts for 80-85% of lung cancers, and the 5-year survival rate remained about 15% and still has not been increased during the last 30 years [3, 4]. As more than 70% NSCLC patients are diagnosed with advanced-stage disease, it is urgent to investigate the molecular mechanism of NSCLC and new targeted therapy in the treatment of advanced NSCLC [5]. Currently, efforts to decipher signaling pathways provide new advances in the understanding of pathogenic mechanism of NSCLC, and microRNAs (miRs) differentially expressed in NSCLC may target molecular pathways involved in carcinogenesis [6–8].

MicroRNA-7 (miR-7), located on human chromosome 9q21, is an important member of the miRNA family and its mature body is composed of 23 nucleotides [9]. Studies have shown that miR-7 was highly expressed in various tissues and organs, and involved in the development of tissues and organs and implicated in the cell proliferation, migration and invasion of various tumors, such as lung cancer, breast cancer, hepatocellular carcinoma and node cancer [10–13]. Focal adhesion kinase (FAK) is a multi-functional regulator of cell signaling within the tumor microenvironment, and controls cell movement, invasion, survival through FAK's kinase-dependent and -independent functions [14]. The extracellular signal-regulated kinase and mitogen-activated protein kinase (ERK/MAPK) signaling pathway has also been implicated in multiple cellular processes such as proliferation, migration and apoptosis, and ERK phosphorylation is regulated by FAK [15–17]. miR-7 has been reported to suppress the key numbers of cancer cell signaling pathway, including EGFR/Ras/Raf/MEK/ERK1/2, EGFR/PI3K/Akt/mTOR, IGF1R/IRS, Integrin/FAK, Rac1/Pak1 and Ack1-mediated signaling transduction, which may implicated in tumorigenesis, progression and metastasis [18]. Based on previous findings, miR-7 targets paired box 6 (Pax6), and can promote NSCLC cell proliferation and invasion via activating the ERK/MAPK signaling pathways by negatively regulating Pax6 protein expression [19]. Furthermore, miR-7 partially targets FAK and acted a key player in the integrin beta (1)-FAK signaling axis, which has been demonstrated in the control of the proliferation of micrometastatic cancer cells disseminated in the lungs [20, 21]. Therefore, we hypothesized that miR-7 may affect cell proliferation, migration and invasion of NSCLC cells through ERK/MAPK signaling pathways. In the present study, we aimed to investigate the potential roles of miR-7 and its target gene FAK mediated by ERK/MAPK signaling pathway in the regulation of cell proliferation, migration and invasion in NSCLC cells.

## RESULTS

### Correlations of clinicopathologica features of NSCLC patients with the expression of miR-7 and the mRNA and protein expressions of FAK and ERK/MAPK signaling pathway-related proteins

**Figure 1:**
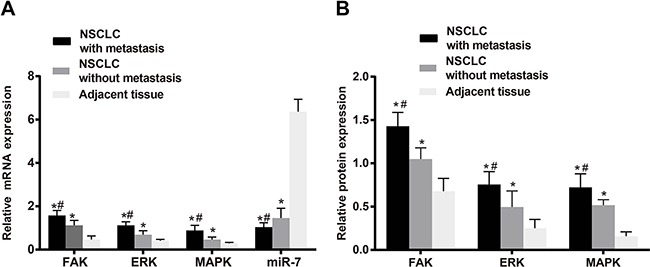
The miR-7 expression and relative mRNA and protein expressions of FAK, ERK and MAPK in NSCLC tissues and the adjacent normal tissues; **A.** miR-7 expression and relative mRNA expressions of FAK, ERK and MAPK in metastatic NSCLC tissues, non-metastatic NSCLC tissues and the adjacent normal tissues; **B.** protein expressions of FAK, ERK and MAPK in metastatic NSCLC tissues, non-metastatic NSCLC tissues and the adjacent normal tissues. Note: *, compared with the adjacent normal tissues, *P* < 0.05; #, compared with non-metastatic NSCLC tissues, *P* < 0.05. NSCLC, non-small cell lung cancer; FAK, focal adhesion kinase; ERK, extracellular regulated protein kinases; MAPK, mitogen-activated protein kinase.

As shown in Figure 1, the expression of miR-7 in adjacent normal tissues was higher that in NSCLC tissues (*P* < 0.05). While higher mRNA and protein expressions of FAK were found in NSCLC tissues in comparison with adjacent normal tissues (both *P* < 0.05). Similarly, the mRNA and protein expressions of ERK and MAPK in NSCLC tissues were higher than those in adjacent normal tissues (all *P* < 0.05). As shown in Table 1, the expressions of miR-7, FAK, ERK and MAPK showed no associations with gender, age, tumor location, tumor size or histological type in NSCLC patients (all *P* > 0.05). However, the expressions of miR-7, FAK, ERK and MAPK were associated with LNM and TNM stage of NSCLC patients (all *P* < 0.05).

**Table 1:** Clinicopathological factors of NSCLC patients and expressions of miR-7 and its downstream proteins

Clinicopathological factors	N	MiR-7	FAK mRNA	ERK mRNA	MAPK mRNA	FAK protein expression	ERK protein expression	MAPK protein expression
Gender								
Male	107	1.206 ± 0.39	1.370 ± 0.314	0.939 ± 0.277	0.696 ± 0.294	1.276 ± 0.231	0.637 ± 0.233	0.628 ± 0.166
Female	53	1.220 ± 0.394	1.423 ± 0.332	0.939 ± 0.273	0.738 ± 0.265	1.255 ± 0.254	0.665 ± 0.149	0.651 ± 0.154
*P*		0.833	0.325	1.000	0.396	0.612	0.413	0.397
Age (years)								
< 50	45	1.189 ± 0.398	1.350 ± 0.340	0.937 ± 0.295	0.747 ± 0.301	1.282 ± 0.221	0.659 ± 0.197	0.641 ± 0.169
≥ 50	115	1.219 ± 0.389	1.402 ± 0.313	0.940 ± 0.268	0.697 ± 0.278	1.264 ± 0.245	0.641 ± 0.213	0.633 ± 0.160
*P*		0.671	0.361	0.936	0.333	0.674	0.629	0.801
LNM								
Positive	93	1.035 ± 0.206	1.579 ± 0.229	1.121 ± 0.162	0.886 ± 0.238	1.427 ± 0.160	0.755 ± 0.150	0.721 ± 0.157
Negative	67	1.456 ± 0.450	1.122 ± 0.224	0.688 ± 0.186	0.467 ± 0.112	1.049 ± 0.130	0.496 ± 0.185	0.516 ± 0.064
*P*		< 0.0001	< 0.0001	< 0.0001	< 0.0001	< 0.0001	< 0.0001	< 0.0001
TNM stage								
I/II	81	1.361 ± 0.470	1.203 ± 0.282	0.773 ± 0.256	0.535 ± 0.208	1.106 ± 0.177	0.544 ± 0.204	0.544 ± 0.106
III/IV	79	1.057 ± 0.191	1.578 ± 0.235	1.110 ± 0.167	0.890 ± 0.237	1.436 ± 0.167	0.751 ± 0.155	0.729 ± 0.157
*P*		< 0.0001	< 0.0001	< 0.0001	< 0.0001	< 0.0001	< 0.0001	< 0.0001
Tumor location								
Left	61	1.248 ± 0.375	1.363 ± 0.308	0.942 ± 0.292	0.729 ± 0.314	1.276 ± 0.243	0.670 ± 0.178	0.657 ± 0.159
Right	99	1.188 ± 0.400	1.403 ± 0.327	0.938 ± 0.265	0.699 ± 0.266	1.264 ± 0.237	0.632 ± 0.225	0.622 ± 0.163
*P*		0.347	0.444	0.920	0.490	0.763	0.267	0.185
Tumor size								
< 5 cm	93	1.176 ± 0.357	1.326 ± 0.275	0.884 ± 0.275	0.659 ± 0.320	1.199 ± 0.258	0.599 ± 0.214	0.596 ± 0.147
≥ 5 cm	67	1.259 ± 0.43	1.426 ± 0.34	0.973 ± 0.27	0.742 ± 0.257	1.312 ± 0.216	0.675 ± 0.201	0.659 ± 0.167
*P*		0.189	0.520	0.943	0.993	0.799	0.778	0.619
Histological type								
Squamous cell carcinoma	80	1.209 ± 0.400	1.407 ± 0.302	0.952 ± 0.255	0.711 ± 0.290	1.261 ± 0.242	0.653 ± 0.223	0.628 ± 0.168
Adenocarcinoma	62	1.201 ± 0.368	1.376 ± 0.331	0.932 ± 0.287	0.710 ± 0.283	1.274 ± 0.237	0.642 ± 0.200	0.640 ± 0.159*
others	18	1.254 ± 0.438	1.381 ± 0.321	0.944 ± 0.299	0.672 ± 0.265	1.236 ± 0.222	0.632 ± 0.174	0.664 ± 0.191
*P*		0.879	0.264	0.587	0.574	0.819	0.387	0.662

NSCLC, non-small-cell lung carcinoma; miR-7, microRNA-7; FAK, focal adhesion kinase; ERK, extracellular signal-regulated kinase; MAPK, mitogen-activated protein kinase; LNM, lymph node metastasis; TNM, tumor node metastasis;

### MiR-7 expression in A549, H1299, H1355 and MRC5 cell lines

**Figure 2:**
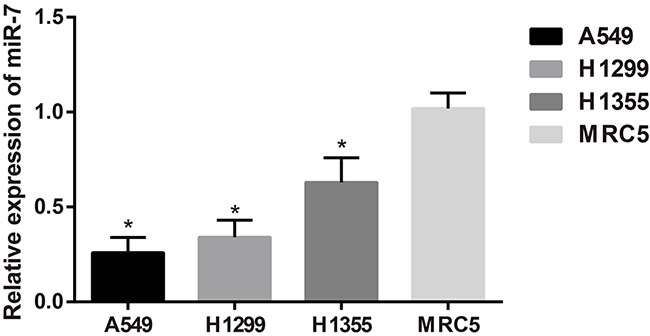
The miR-7 expression in A549, H1299, H1355 and MRC5 cell lines; *, compared with the MRC5 cell line, *P* < 0.05.

The qRT-PCR results showed that the expressions of miR-7 in A549, H1299 and H1355 cell lines were lower than that in MRC5 cell line (all *P* < 0.05). As a result, miR-7 was lowly expressed in NSCLC tissues and cell line. As shown in Figure 2, miR-7 was lowly expressed in A549 and H1299 cells, and thus A549 and H1299 cell lines were used for the following studies.

### MiR-7 and FAK mRNA expressions in A549 and H1299 cell lines after transfection

**Figure 3:**
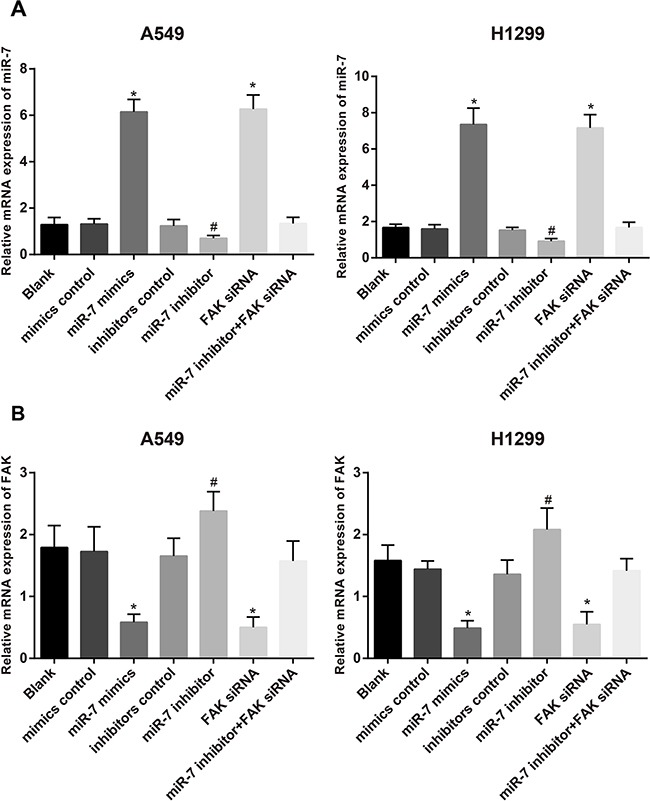
The expressions of miR-7 **A.** and *FAK* mRNA **B.** in A549 and H1299 cells in blank group, miR-7 mimic control group, miR-7 mimic group, inhibitor control group, miR-7 inhibitor group, FAK siRNA group and miR-7 inhibitor + FAK siRNA group detected by qRT-PCR; *, compared with the mimics control group, *P* < 0.05; #, compared with inhibitors control group, *P* < 0.05. FAK, focal adhesion kinase; miR-7, microRNA-7.

The results of qRT-PCR showed that in A549 and H1299 cell lines, no distinct difference was found in the expressions of miR-7 and *FAK* mRNA among mimics control group, inhibitors control group, miR-7 inhibitor + FAK siRNA group and blank group (all *P* > 0.05). Compared with the mimics control group, significantly increased miR-7 expression and decreased FAK mRNA expression were found in the miR-7 mimics group (both *P* < 0.05). No significant differences on the expressions of miR-7 and FAK mRNA were found between FAK siRNA group and miR-7 mimics group (all *P* < 0.05). In addition, miR-7 inhibitors group had visibly decreased miR-7 expression and increased FAK mRNA expression, as compared to the miR-7 inhibitors control group, (both *P* < 0.05) (Figure 3).

### Targeting relationship between miR-7 and FAK

**Figure 4:**
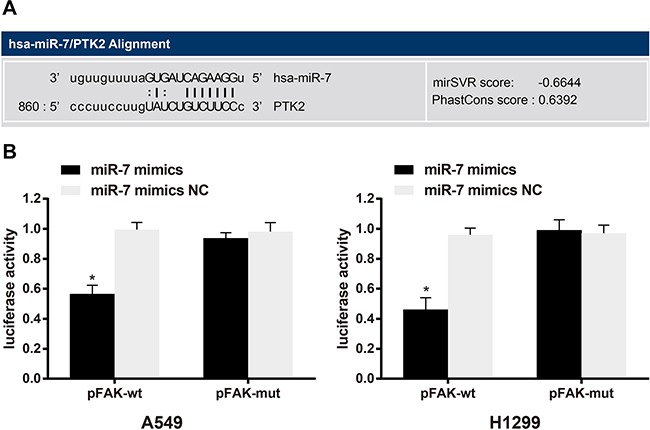
**MicroRNA**.org predicted that *FAK/PTK2* is the target gene for miR-7 **A.** Dual luciferase reporter gene assay confirmed that *FAK/PTK2* is the target gene of miR-7 in A549 cells and H1299 cells **B.** Note: *, compared with the miR-7 mimics NC group, *P* < 0.05. FAK, focal adhesion kinase 1; PTK2, protein tyrosine kinase 2; NC, negative control; miR-7, microRNA-7

Biological prediction website (www.microRNA.org) showed that miR-7 is able to target *FAK* (Figure 4A). In order to confirm that *FAK* is a direct target gene of miR-7, luciferase reporter vector recombinant plasmid pFAK-Wt and pFAK-Mut were constructed based on *FAK* mRNA 3′-UTR. The dual luciferase reporter gene assay indicated that, A549 cells, the luciferase activity in the miR-7 mimics + pFAK-Wt group decreased by about 42%, compared with other groups (all *P* < 0.05). In H1299 cells, the luciferase activity in the miR-7 mimics + pFAK-Wt group decreased by about 53%, compared with other groups (all *P* < 0.05) (Figure 4B). While no significant differences were found in the luciferase activity in each group of *FAK* mut-3′-UTR (all *P >* 0.05). Thus, *FAK* is the potential target gene of miR-7.

### The protein expressions of FAK, ERK and MAPK in A549 and H1299 cell lines after transfection

**Figure 5:**
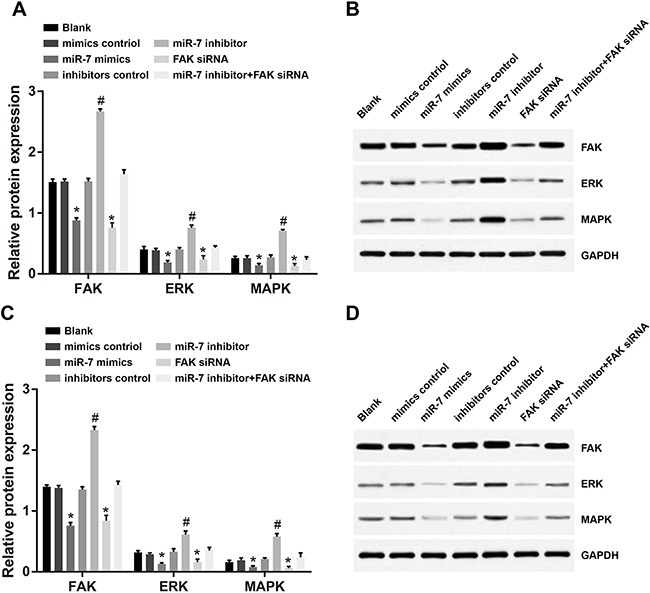
Comparison of protein expressions of FAK, ERK and MAPK in A549 and H1299 cells detected by Western-Blotting (**A.** protein expressions of FAK, ERK and MAPK in A549 cells; **B.** Western-Blotting results in A549 cells; **C.** protein expressions of FAK, ERK and MAPK in H1299 cells; **D.** Western-Blotting results in H1299 cells); *, miR-7 mimics group compared with the mimics control group, *P* < 0.05; #, miR-7 inhibitor group compared with the inhibitors control group, *P* < 0.05. FAK, focal adhesion kinase; ERK, extracellular regulated protein kinases; MAPK, mitogen-activated protein kinase; miR-7, microRNA-7

Western-Blotting results showed, in A549 and H1299 cell lines, no distinct difference in protein expressions of FAK and ERK/MAPK among mimic NC group, inhibitor NC group, blank group or miR-7 inhibitor + FAK siRNA group (all *P* > 0.05). Compared to the blank group, the miR-7 mimic group was associated with significantly decreased protein expressions of FAK, ERK and MAPK under the overexpression of miR-7 (all *P* < 0.05). No significant differences in protein expressions of FAK, ERK and MAPK were observed between miR-7 mimics group and FAK siRNA group (all *P* > 0.05). However, miR-7 inhibitor group showed significantly increased protein expressions of FAK, ERK and MAPK as compared to the blank group (all *P* < 0.05) (Figure 5).

### Effects of miR-7 on the proliferation of A549 cells and H1299 cells

**Figure 6:**
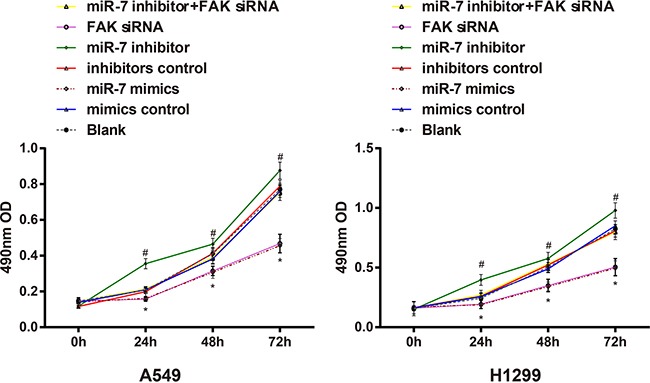
The effect of miR-7 on the cell proliferation of A549 and H1299 cells detected by MTT method; Note: *, compared with the mimics control group at the same point, *P* < 0.05; #, compared with the inhibitors control group at the same time point, *P* < 0.05. MiR-7, microRNA-7; MTT, methyl thiazolyl tetrazolium.

The OD values measured in A549 cells and H1299 cells after transfection were shown in Figure 6. The results showed that A549 cells and H1299 cells transfected with miR-7 mimics had significantly slower growth rate than cells in mimic control group (both *P* < 0.05). The growth rate of miR-7 mimics group and FAK siRNA group had no significant difference (*P* > 0.05); no significant differences on the growth rate were observed among blank group, mimics control group, inhibitors control group and miR-7 inhibitor + FAK siRNA group (all *P* > 0.05). While the growth rate of miR-7 inhibitors group was faster than that of the miR-7 inhibitors control group (*P* < 0.05).

### Effect of miR-7 on the migration of A549 cells and H1299 cells

**Figure 7:**
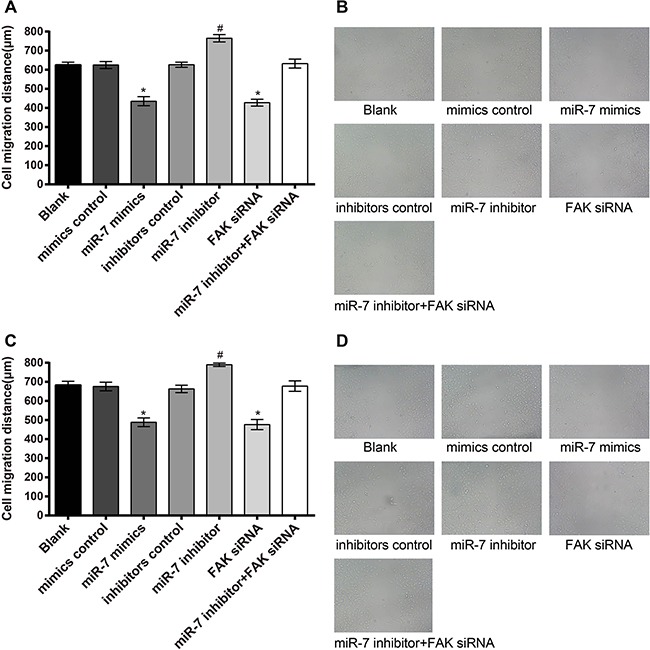
The results of the A549 and H1299 cells migration **A.** comparison of scratch width of A549 cells among each group; **B.** wound scratch assay results of A549 cells under light microscope; **C.** comparison of scratch width of H1299 cells among each group; **D.** wound scratch assay results of H1299 cells under light microscope; *, miR-7 mimics group compared with the mimics control group, *P* < 0.05; #, miR-7 inhibitor group compared with the inhibitors control group, *P* < 0.05. MiR-7, microRNA-7; MTT, methyl thiazolyl tetrazolium.

As shown in Figure 7, migration distance of A549 cells in miR-7 mimics group was (435.22 ± 23.83) μm, blank group (625.45 ± 14.41) μm, miR-7 inhibitor group (765.15 ± 19.49) μm, mimics control group (624.70 ± 18.22) μm, inhibitors control group (626.32 ± 13.12) μm, FAK siRNA group (427.49 ± 17.75) μm and miR-7 inhibitor + FAK siRNA group (632.37 ± 23.38) μm. These results showed that there was no significant difference in migration distance of A549 cells between blank group and mimics control group, inhibitors control group and miR-7 inhibitor + FAK siRNA group (all *P* > 0.05). Compared to the mimics control group, miR-7 mimic group showed significantly reduced cell migration ability (*P* < 0.05), while cell migration ability was enhanced significantly in the miR-7 inhibitor group as compared to the inhibitors control group (*P* < 0.05). No significant difference was observed between FAK siRNA group and miR-7 mimics group (*P* > 0.05). In H1299 cells, migration distance in miR-7 mimics group was (488.26 ± 22.65) μm, blank group (683.74 ± 19.52) μm, miR-7 inhibitor group (789.41 ± 8.90) μm, mimics control group (675.83 ± 22.78) μm, inhibitors control group (662.76 ± 19.30) μm, FAK siRNA group (476.52 ± 26.63) μm and miR-7 inhibitor + FAK siRNA group (677.72 ± 27.56) μm. Similar results of H1299 cells were observed on the migration distance in each group as in A549 cells.

### Effect of miR-7 on the invasion of A549 cells and H1299 cells

**Figure 8:**
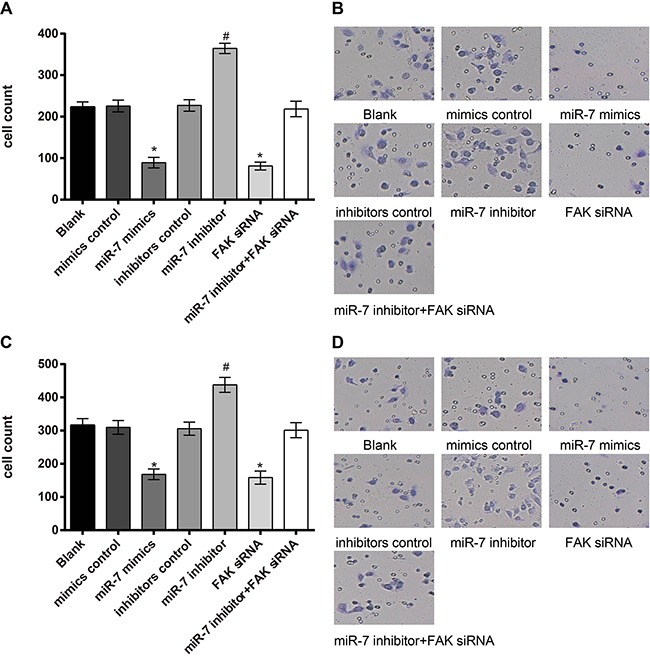
A549 and H1299 cells invasion ability detected by Transwell assay **A.** comparison of the invasive cell numbers of A549 cells in each group; **B.** Transwell assay results of A549 cells under light microscope; **C.** comparison of invasive cell numbers of H1299 cells among each group; **D.** Transwell assay results of H1299 cells under light microscope; *, miR-7 mimics group compared with the mimics control group, P < 0.05; #, miR-7 inhibitor group compared with the inhibitors control group, P < 0.05.

As shown in Figure 8, invasive cell numbers of A549 cells in miR-7 mimics group were (89.08 ± 12.56), blank group (223.51 ± 11.89), miR-7 inhibitor group (364.83 ± 12.24), mimics control group (225.53 ± 14.08), inhibitors control group (227.02 ± 13.92), FAK siRNA group (81.16 ± 9.64) and miR-7 inhibitor + FAK siRNA group (218.41 ± 18.65). There was no visible difference in invasive cell numbers between blank group and mimics control group, inhibitors control group and miR-7 inhibitor + FAK siRNA group (all *P* > 0.05). Compared to the mimics control group, cell invasion ability was significantly decreased in the miR-7 mimics group, while cell invasion ability was significantly enhanced in the miR-7 inhibitor group, compared with inhibitors control group (both *P* < 0.05). No visible difference in invasive cell numbers was found between FAK siRNA group and miR-7 mimics group (*P* > 0.05). In the miR-7 mimics group, invasive cell numbers were reduced due to diminished cell invasion ability, and cells were widely spaced; contrarily, close cell distribution and larger invasive cell numbers were found in the miR-7 inhibitors group. In H1299 cells, invasive cell numbers in miR-7mimics group were (168.27 ± 15.71), blank group (316.56 ± 19.41), mimics control group (309.64 ± 20.67), inhibitors control group (305.85 ± 19.72), miR-7 inhibitor group (437.70 ± 22.33), FAK siRNA group (158.43 ± 19.79) and miR-7 inhibitor + FAK siRNA group (301.17 ± 22.54). Similar results of H1299 cells were observed on the invasive cell numbers in each group as in A549 cells.

## DISCUSSION

MiR-7 is implicated in the development and progression of tumors, and can inhibit the occurrence of many tumors and metastasis. In the present study, we aimed to explore the potential roles of miR-7 in NSCLC cells and in FAK mediated ERK/MAPK signaling pathway through a series of experiments to inhibit the expression of ERK/MAPK in the regulation of the FAK pathway. We found that the miR-7 was lower expressed in the NSCLC tissues and NSCLC cell lines, but the expression levels of FAK, ERK and MAPK was up-regulated. Dual luciferase reporter gene assay results also showed that *FAK* is a direct target gene of miR-7. Further, miR-7, FAK, ERK and MAPK expressions were associated with LNM and TNM stages in NSCLC patients. These results suggested that the down-regulated expression of miR-7 may be implicated in the development and progression of NSCLC by up-regulating the expression of FAK mediated ERK/MAPK signaling pathway.

MiR-7 is a putative tumor suppressor in various solid tumors, and is reported to be down-regulated in NSCLC, which may suppress tumorigenesis by targeting a number of important proto-oncogenes and by inhibiting EGFR/AKT pathway activation [22, 23]. It has been reported that downexpression of miR-7 in NSCLC may be implicated in promoting cancer cell progress, and consequently results in NSCLC growth [19, 24], which were in line with our study results. In addition, miR-7 is a tissue-specific miR to be directly involved in NSCLC [25]. Low expression level of miR-7 was significantly correlated with LNM in patients with cervical cancer [26]. We found that expressions of miR-7, FAK, ERK and MAPK are associated with LNM and TNM stages in NSCLC patients, posing a potential role of miR-7 and its downstream pathway as non-invasive biomarkers for assessing NSCLC clinical outcome.

To better understand the potential molecular mechanism of miR-7 on the development of NSCLC, we conducted *in vitro* study based on the A549 and H1299 NSCLC cell lines. In the current study, we found that miR-7 can inhibit FAK protein expression, while the expression of FAK is positively associated with the expressions of ERK and MAPK, indicating that miR-7 inhibits ERK/MAPK signaling pathway by targeting FAK. And also, the miR-7 can inhibit NSCLC cells migration and invasion, and elevated miR-7 expression may enhance the inhibiting ability, which may decrease migration and invasion ability of NSCLC cells. We suspected that the migration and invasion ability may be associated with the negative regulation of miR-7 ERK/MAPK signaling pathway by targeting FAK.

Extensive roles of miR-7 in regulating cancer cell initiation, proliferation, migration, invasion, survival and death by targeting a number of oncogenic signaling pathways have been reported [27, 28]. Partially consist with the role of miR-7 in down-regulating FAK and inhibiting the ERK/MAPK signaling pathway, miR-7 overexpression is associated with suppressed proliferation, cell migration and tumorigenicity, and induced cell apoptosis in NSCLC cells [29]. Further, Hao et al [21], Kong et al [30] and Wu et al [31] also indicated that, via targeting FAK expression, miR-7 is capable of inhibiting metastasis and invasion in cervical cancer, inhibiting epithelial-to-mesenchymal transition (EMT) and metastasis of breast cancer cells, and regulating glioblastoma cell invasion. Important findings in our study also showed that significantly increased miR-7 and decreased FAK mRNA expressions, decreased protein expressions of FAK, ERK and MAPK, and inhibited cell proliferation, migration and invasion were found in miR-7 mimic group; while opposite regarding miR-7 inhibitor group. Combining these significant results in the study, we support the hypothesis that miR-7 may inhibit the ERK/MAPK signaling pathway by down-regulating the targeted FAK, which can inhibit cell proliferation, migration and invasion in NSCLC cells. As further extended by a recent study, miR-7 presents biological functions in NSCLC acting as a useful mediator in suppressing growth and inducting apoptosis in NSCLC cells [32]. A precious study revealed that miR-7 is identified to directly down-regulate FAK expression, and miR-7 inhibits EMT and metastasis of cancer cells through targeting FAK [30]. FAK may mediate cell signaling transduction in the occurrence and development of tumors, and then induce the cell adhesion, differentiation, proliferation and migration, which may be implicated in the tumor formation, invasion, metastasis and clinical prognosis of cancer patients [33]. Han et al. have demonstrated that FAK expression was up-regulated in the NSCLC tissues and was closely related to tumor cell proliferation and metastasis [34]. Furthermore, Luo and his colleagues have revealed that the ERK/MAPK signaling pathways were down-regulated in the miR-7-overexpressed NSCLC A549 cells [19].

In conclusion, miR-7 was lower expressed in NSCLC tissues and NSCLC cell lines and associated with LNM and TNM stages of NSCLC patients, indicating that the down-regulated expression of miR-7 may be implicated in the development and progression of NSCLC. For the potential molecular mechanisms, the miR-7 can inhibit the activation of ERK/MAPK signaling pathway by down-regulating FAK expression, thereby suppressing the proliferation, migration and invasion of NSCLC cells. The miR-7 and its target gene FAK may be novel targets for the diagnosis and treatment of NSCLC.

## MATERIALS AND METHODS

### Study subjects

Tissue specimens from surgically treated NSCLC patients (n = 160; mean age, 57.5 ± 14.2 years; male, 107; female, 53) in Affiliated Changzhou No.2 People's Hospital were collected from August 2012 to November 2015. All patients received no radiotherapy or chemotherapy before operation. NSCLC tissues and adjacent normal tissues were resected during operation, a part of tissue specimens were immediately placed and frozen in liquid nitrogen, and stored at -80°C; another part of tissue specimens were fixed in formalin and well-marked. All the specimens were confirmed by pathological diagnosis. NSCLC TNM staging [35]: stage I/II (n = 81) and stage III/IV (n = 79). Lymph node metastasis (LNM): positive (n = 93) and negative (n = 67). Tumor site: left lung (n = 61) and right lung (n = 99). Tumor size: > 5 cm (n = 67) and < 5 cm (n = 93). Histological type: squamous cell carcinoma (n = 80) and adenocarcinoma (n = 62) and others (n = 18). This study protocol was approved by the Ethics Committee of Affiliated Changzhou No.2 People's Hospital, and all patients signed informed consent. Quantitative real-time polymerase chain reaction (qRT-PCR) and Western-Blotting were used to verify the expressions of miR-7 and the mRNA and protein expressions of FAK and ERK/MAPK signal pathway-related proteins.

### Cell culture, transfection and grouping

NSCLC cell lines, including A549, H1299 and H1355, and a normal human fetal lung fibroblast cell line (MRC-5) were obtained from Shanghai Cell Bank of Chinese Academy of Sciences, China. All cell lines were cultured in RPMI 1640 medium (Gibco, Grand Island, New York, USA) containing 10% fetal bovine serum (FBS) (Hyclone, Logan, UT, USA), and incubated in a 5% CO_2_ incubator at 37°C. Cells in logarithmic phase were collected for transfection. NSCLC cell lines with the lowest expression of miR-7 were used for further study. NSCLC cells in the logarithmic growth phase were assigned into 7 groups: blank group (non-transfected group); miR-7 mimics group (transfected with miR-7 mimics); miR-7 mimics control group (transfected with miR-7 mimic negative control [NC] sequence); miR-7 inhibitors group (transfected with miR-7 inhibitors); miR-7 inhibitors control group (transfected with miR-7 inhibitor NC sequence); FAK siRNA group (transfected with FAK siRNA plasmid); miR-7 inhibitors + FAK siRNA group (co-transfected with miR-7 inhibitors and FAK siRNA plasmid). All the miR-7 mimics, miR-7 mimic NC sequence, miR-7 inhibitors, miR-7 inhibitor NC sequence and FAK siRNA were purchased from Shanghai GenePharma Co.,Ltd (Shanghai, China). After 24 h cell transfection, the total RNA was extracted for qRT-PCR to detect relative expressions of miR-7 in the transfected cells; and after 72 h cell transfection, the total protein was extracted and the protein expressions of FAK, MAPK and ERK were detected by Western-Blotting (Table 2).

**Table 2:** Experiment related miR sequences

	Sequences
MiR-7 mimic	5′-UGGAAGACUAGUGAUUUUGUUGU-3′
Mimic control	5′-UGGAAGACUUGUGAUUUUGUUGU-3′
MiR-7 inhibitor	5′-ACAACAAAAUCACUAGUCUUCCA-3′
Inhibitor control	5′-ACAACAAAAUCACAAGUCUUCCA-3′

MiR, microRNA

### *FAK* siRNA plasmid construction

According to the *FAK* mRNA (GI:439874) in GeneBank and based on the designed rules of siRNA, a pair of specific siRNA sequences for blocking FAK mRNA were designed through a siRNA design tool (American Ambion Company). The enzyme restriction sites, *BamH I* and *Hind III*, were added to the two ends of the siRNA sequences, and TTTT termination signal and primer structure BamH I-Sense-Loop-Antisense-termination signal -Sal I Hind III were added before the 3′-terminal restriction sites. Positive-sense strand was: 5′-GATCCCCACCTGGGCCAGTATTATTTCAAGACGATAATACTGGCCCAGGTGGTTTTTTGTCGACA-3′; and the antisense strand was: 3′-GCGGTGGACCCGGTCATAATAAAGTTCTGCTATTATGACCGGGTCCACCAAAAAACAGCTGTTCGA-5′. The plasmids were synthesized by Wuhan Genesil Biotechnology Co., Ltd (Wuhan, China). Constructed plasmids were transformed into Escherichia coli DH5α, and then screened by a plate containing kanamycin. Positive colonies were extracted and identified by enzyme digestion, and sequencing analysis was conducted by Key Laboratory of Molecular Biology on Infectious Disease of Jilin University. The recombinant plasmids were named as FAK siRNA. Sequencing analysis results demonstrated that plasmids were constructed successfully.

### RNA extraction and qRT-PCR

The total RNA was extracted from A549, H1299, H1355 and MRC5 cells, NSCLC tissues and adjacent normal tissues by Trizol reagent. The purity and concentration of RNA were determined by ultraviolet (UV) spectrophotometer, and the integrity of RNA was observed by agarose gel electrophoresis. Primescript™ RT reagent Kit (Takara biotechnology (Dalian) CO., LTD.) was used for reverse transcription, SYBR^®^ premix Ex Taq™ qRT-PCR kit was used for PCR amplification, and amplified primer sequences are shown in Table 3. Glyceraldehyde-3-phosphate dehydrogenase (GAPDH) was used as reference gene. PCR results were analyzed using Opticon Monitor 3 software (Bio-Rad). All data were analyzed by adopting 2^−ΔΔCt^ method, with 2^−ΔΔCt^ demonstrating the relative expression ratios of the target gene of the case group to the control group (ΔΔCT = ΔCt _case group_ - ΔCt _control group_, ΔCt = Ct _miR_ - Ct_GAPDH_), Ct represents threshold cycle. The experiment was triplicate.

**Table 3:** PCR primers and probe sequences

Primers	Sequences
MiR-7	
Upstream	5′-ACGCGGCAAGATGCTGGCA-3′
Downstream	5′-CAGTGCTGGGTCCGAGTGA-3′
Probe	5′-FAM-TCGTATCCAGTGCGAATGACTC-TAMRA-3′
FAK	
Upstream	5′-CAAGGCAGAAGGCAGCTATG-3′
Downstream	5′-ATGGGAGGGAGCGTTTGA-3′
ERK	
Upstream	5′-AGTCGGAGTAGGGTGTTTCG-3′
Downstream	5′-TTTTGCTTCTGGATGTGGG-3′
MAPK	
Upstream	5′-AAGTCACGCTGGAGTGGTTC-3′
Downstream	5′-CCCCGTCATCAGTTCATACAA-3′
GAPDH	
Upstream	5′-TCCGGGTGATGCTTTTCCTAG-3′
Downstream	5′-TTTGCGGTGGAAATGTCCTTTTC-3′

MiR-7, microRNA-7; FAK, focal adhesion kinase; ERK, extracellular signal-regulated kinase; MAPK, mitogen-activated protein kinase; GAPDH, glyceraldehyde-3-phosphate dehydrogenase; PCR, polymerase chain reaction;

### Dual luciferase reporter gene assay

MicroRNA.org (http://www.microrna.org) was used for miR-7 target prediction, and to verify whether *FAK* is a direct target gene of miR-7. Full-length 3′-untranslatedregion (3′-UTR) of the *FAK* gene was amplified, cloned and sequenced. PCR products were cloned into multiple cloning sites of PmirGLO (Promega Corp., Madison, WI, USA) luciferase gene downstream. The binding sites of miR-7 and target genes were subjected to site-specific mutagenesis, and pRL-TK vector expressing Renilla luciferase (TaKaRa) was used as internal reference to adjust differences in cell numbers and transfection efficiency. MiR-7 mimics and miR-7 mimic NC were respectively co-transfected with luciferase reporter vector into NSCLC cells. Dual luciferase activity was detected based on the Promega kit.

### Western-blotting

The transfected cells were collected, washed in phosphate buffered saline (PBS) twice, lysed in 100 μl cell lysate (4°C, 30 min) and centrifuged (12000 g, 10 min) to obtain the supernatant, and the protein concentration was determined by Bradford method. The total proteins (50 μg) were run on 12% SDS-PAGE and electro-transferred to polyvinylidene fluoride (PVDF) membranes, membranes were blocked with 5% skim milk at room temperature for 1 h, incubated with polyclonal antibodies: rabbit anti human FAK (ab40794; 1:1000; Abcam Company), MAPK (ab197348; 1:500; Abcam Company) and ERK (ab17942, 1:1000; Abcam Company) and mouse anti human GAPDH monoclonal antibody (ab8245; 1:500; Abcam Company) overnight at 4 °C, washed 3 times to remove primary antibodies, further incubated with IRDye™ 700DX labeled sheep anti rabbit IgG (ab6721, 1:2000; Abcam Company; for the detection of FAK, MAPK, ERK) and IRDye™ 800DX labeled sheep anti mouse IgG (ab6789, 1:2000; Abcam Company; for the detection of GAPDH) at room temperature in the dark for 1 h, the membrane was fully washed, and scanned with the Odyssey Infrared Imaging System (LI-COR Biosciences,Lincoln, NE). The integrated optical density (IOD) value of each band was calculated, and the relative expressions of the target proteins were calculated by the ratio of the IOD value of the target band and the IOD value of the corresponding internal reference (*β*-actin).

### Methyl thiazolyl tetrazolium (MTT)

After 48 h transfection, cells in each group were counted and were inoculated in four 96-well plates (density, 2 × 10^2^, 200 μl per well, 8 repeated wells). MTT solution (20 μl) was added into each well for 4 h incubation at 37°C, then the incubation was terminated, and culture supernatant was discarded. Dimethyl sulfoxide (DMSO) (150 μl) (Sigma, Englewood Cliffs, NJ, USA) is added to each well, and gently shaken for 10 min in an enzyme-linked immunosorbent detector. Absorbance values (OD) were determined at a wavelength of 490 nm at 0 h, 24 h, 48 h and 72 h, respectively. Cell viability curve was drawn with time as the X-axis, the OD value as the Y-axis.

### Wound scratch assay

Cell migration was assessed using a wound scratch assay. NSCLC cells were digested, and cell density was adjusted to 5 × 10^5^/mL. NSCLC cells were seeded in Metrigel coated 96-well plates, and subjected to routine culture to form cell monolayers. The cell layers were vertically scratched and rinsed once, and the primary medium was replaced by RPMI 1640 medium containing 10 g/L bovine serum albumin (BSA) and 1% FBS. The scratch area distance was measured under a microscope. After 24 h culture, additional replacement was performed with RPMI 1640 medium containing 10% FBS. After the additional 24 h culture, the relative distance of cells migrated to the scratched area was measured under the microscope.

### Transwell assay

After 24 h transfection, NSCLC cells were cultured in serum free medium for 12 h, digested, washed twice with PBS, and suspended in serum free medium Opti-MEMI (Invitrogen) containing 10 g/L BSA. The cell density was adjusted to 3 × 10^4^/ml. The experiment was performed in 24-well 8 μm Transwell plates (Corning-Costar, Corning, NY, USA) (3 chambers per group; 100 μl cell suspension per chamber). The lower chamber with the addition of 600 μL 10% RPMI1640 medium was incubated in 5%CO_2_ under 37°C. In migration test: 24 h later, cells were fixed with 4% paraformaldehyde for 30 min, and the chambers were subjected to 0.2% Triton X-100 (sigma, USA) solution for 15 min and 0.05% gentian violet dye for 5 min. In invasion test: 50 μl Matrigel (sigma) was spread in the chambers before the invasion test, and 48 h later, the chambers were fixed and stained by the above-mentioned method. The number of stained cells was counted under inverted microscope. Five field counts were randomly selected. Three independent experiments were performed.

### Statistical analysis

SPSS 19.0 software (IBM Corporation, NY, USA) was applied to statistical analysis. Continuous variables were presented as means ± standard deviation (SD), and comparisons were performed with *t*-test, one-way ANOVA and least significant difference t (LSD-t) if appropriate. Categorical variables were as frequencies and percentages, and comparisons were performed with chi-square test. A two-tailed *P* value of < 0.05 indicates statistically significant.
